# Increased Histone Acetylation and Decreased Expression of Specific Histone Deacetylases in Ultraviolet-Irradiated and Intrinsically Aged Human Skin In Vivo

**DOI:** 10.3390/ijms22042032

**Published:** 2021-02-18

**Authors:** Yuri Lee, Mi Hee Shin, Min-Kyoung Kim, Yeon Kyung Kim, Hye Sun Shin, Dong Hun Lee, Jin Ho Chung

**Affiliations:** 1 Department of Dermatology, Seoul National University College of Medicine, Seoul 03080, Korea; lepommier@snu.ac.kr (Y.L.); fiona1@hanmail.net (M.H.S.); mkkimsnu@gmail.com (M.-K.K.); duriyk@hanmail.net (Y.K.K.); shs6211@snu.ac.kr (H.S.S.); 2 Department of Biomedical Sciences, Seoul National University Graduate School, Seoul 03080, Korea; 3 Institute of Human-Environment Interface Biology, Medical Research Center, Seoul National University, Seoul 03080, Korea; 4 Institute on Aging, Seoul National University, Seoul 03080, Korea

**Keywords:** histone deacetylase, sirtuin, ultraviolet, acetylated histone H3, skin aging

## Abstract

Histone deacetylases (HDACs) are conserved enzymes that remove acetyl groups from lysine side chains in histones and other proteins and play a crucial role in epigenetic regulation. Previously, we showed that histone acetylation is implicated in ultraviolet (UV)-induced inflammation and matrix impairment. To elucidate the histone acetylation status and specific HDACs involved in skin aging, we examined the changes in histone acetylation, global HDAC activity, and the expression of HDACs and sirtuins (SIRTs) in intrinsically aged and photoaged human skin as well as in UV-irradiated human skin in vivo. Following acute UV irradiation, the acetylated histone H3 (AcH3) level was increased, but HDAC activity and the expression levels of HDAC4, HDAC11, and SIRT4 were significantly decreased. In intrinsically aged skin, AcH3 levels were increased, but HDAC activity and the expression levels of HDAC4, HDAC5, HDAC10, HDAC11, SIRT6, and SIRT7 were significantly decreased. However, histone acetylation and HDAC expression in photoaged skin were not significantly different from those in intrinsically aged skin. Collectively, HDAC4 and HDAC11 were decreased in both UV-irradiated and intrinsically aged skin, suggesting that they may play a universal role in increased histone acetylation associated with skin aging.

## 1. Introduction

Skin is a multifunctional organ with diverse regulatory properties and operates as a barrier between the external environment and internal milieu [1,2]. Physiological changes in the skin are affected by intrinsic and extrinsic factors. Ultraviolet (UV) light is the most well-characterized extrinsic factor that aggravates skin aging. UV irradiation not only induces skin cancer, autoimmune responses, and photoaging, but it also regulates global homeostasis via activation of neuroendocrine pathways [3]. Exposure of human skin to UV light modulates the synthesis of extracellular matrix proteins, such as procollagen, elastin, and fibrillin-1. In addition, it induces the expression of matrix metalloproteinases (MMPs), leading to degradation of the extracellular matrix during skin aging [4,5,6,7]. Intrinsically aged skin shows fine wrinkles, decreased elasticity, and epidermal thinning in sun-protected areas, whereas chronic exposure to sunlight triggers photoaging characterized by coarser wrinkles, dyschromia, multiple telangiectasias, and solar elastosis [8,9,10]. Intrinsic aging and photoaging share several molecular changes, such as altered signal transduction pathways that promote the expression of MMPs and decrease the synthesis of procollagen [11]. Recent studies suggest that skin aging-related gene expression is regulated via epigenetic mechanisms, such as chromatin modification and DNA methylation [12,13,14,15].

Epigenetics usually refers to heritable changes in gene expression without any change in the DNA sequence [16]. Epigenetic mechanisms include DNA methylation, histone modification, chromatin remodeling, and RNA interference [17]. Histone modifications include histone methylation, acetylation, ubiquitination, and phosphorylation. The acetylation of core histones is controlled by the opposing actions of histone acetyltransferases (HATs) and histone deacetylases (HDACs), the activities of which are typically correlated with gene activation and gene repression/silencing, respectively [18,19]. These epigenetic mechanisms noticeably influence skin aging. Indeed, in a previous study, we demonstrated that UV irradiation increased the acetylation of histone H3 by decreasing the enzymatic activity of HDAC and increasing the activity of HAT in human dermal fibroblasts [14]. In addition, treatment with a p300HAT inhibitor or knockdown of p300 inhibited UV-induced acetylated histone H3 (AcH3) and MMP-1 expression in human skin fibroblasts [14]. We also found that the epigenetic crosstalk between histone acetylation and DNA methylation plays an important role in regulating collagen transcription induced by UV irradiation in human skin fibroblasts [12].

HDACs are classified into four classes. Class I HDACs include HDAC1, HDAC2, HDAC3, and HDAC8; class II HDACs include HDAC4, HDAC5, HDAC6, HDAC7, HDAC9, and HDAC10; and class IV HDACs include HDAC11. The SIRT1-7 genes are classified as class III HDACs [20]. Although some SIRT genes have been actively investigated with regard to aging and longevity in recent years, changes in the expression of specific HDACs in aged human skin in vivo remain to be elucidated.

In the present study, we investigated the changes in AcH3, global HDAC enzyme activity, and levels of 18 HDACs in UV-irradiated human skin and in intrinsically aged and photoaged human skin in vivo.

## 2. Results

### 2.1. UV-Irradiated Human Skin Shows Increased AcH3 Levels but Decreased Global HDAC Activity and HDAC4, HDAC11, and SIRT4 Expression In Vivo

**Figure 1:**
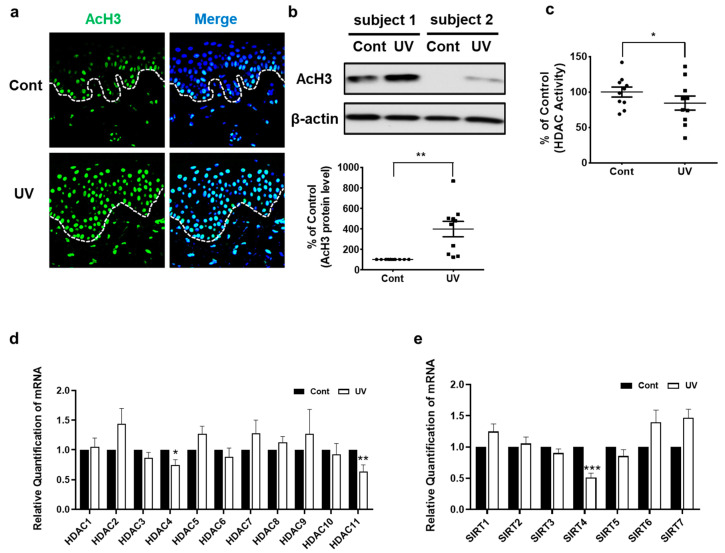
Acetylated histone H3 was increased, but HDAC activity and expression levels of HDAC4, HDAC11, and SIRT4 were decreased in UV-irradiated human skin in vivo. The buttock skin of young subjects was obtained by punch biopsy at 24 h after UV irradiation (two minimal erythema doses (MEDs)). Immunofluorescence analysis of AcH3 and DAPI staining of paraffin-embedded sections (*n* = 6). Original magnification: ×200 (**a**). Western blot analysis of AcH3. The bands are representative of the results obtained for 10 subjects. The bar graph shows densitometric quantification of AcH3 levels. (*n* = 10) (**b**). Total HDAC activity was measured using an HDAC assay kit (*n* = 10) (**c**). Expression levels of HDAC and SIRT mRNAs following UV irradiation were compared with those in non-irradiated control skin by qPCR (*n* = 10) (**d**,**e**). Data were normalized to the control. Data are presented as means ± SEM. Statistical significances were determined using a two-tailed paired *t*-test. * *p* < 0.05, ** *p* < 0.01, *** *p* < 0.001 vs. non-irradiated skin (Cont). Cont: control.

We previously showed that UV irradiation of human dermal fibroblasts increased AcH3 levels and decreased HDAC activity [14]. Here, we first examined whether UV irradiation could affect these epigenetic modifications in human skin in vivo. Immunofluorescence staining showed increased AcH3 expression in both the epidermis and dermis of UV-irradiated human skin, compared with those of non-irradiated skin (Figure 1a). Similarly, Western blot analysis showed that AcH3 levels were significantly increased by an average of 396.8% ± 74.7% in UV-irradiated skin (Figure 1b). On the contrary, global HDAC activity was significantly decreased by an average of 18.7% ± 7.1% in UV-irradiated skin (Figure 1c). In agreement with our previous in vitro data [14], UV irradiation resulted in significantly increased AcH3 levels and decreased global HDAC activity in human skin in vivo.

Next, we investigated changes in the expression of 11 HDACs and 7 SIRTs upon acute UV irradiation of human skin in vivo. The expression of HDAC4, HDAC11, and SIRT4 mRNA was significantly decreased by an average of 24.9%, 35.9%, and 49.1%, respectively, compared to that in non-irradiated control skin (Figure 1d,e).

### 2.2. Intrinsically Aged Human Skin Shows Increased AcH3 Levels but Decreased Global HDAC Activity and HDAC4, HDAC5, HDAC10, HDAC11, SIRT6, and SIRT7 Expressions In Vivo

**Figure 2:**
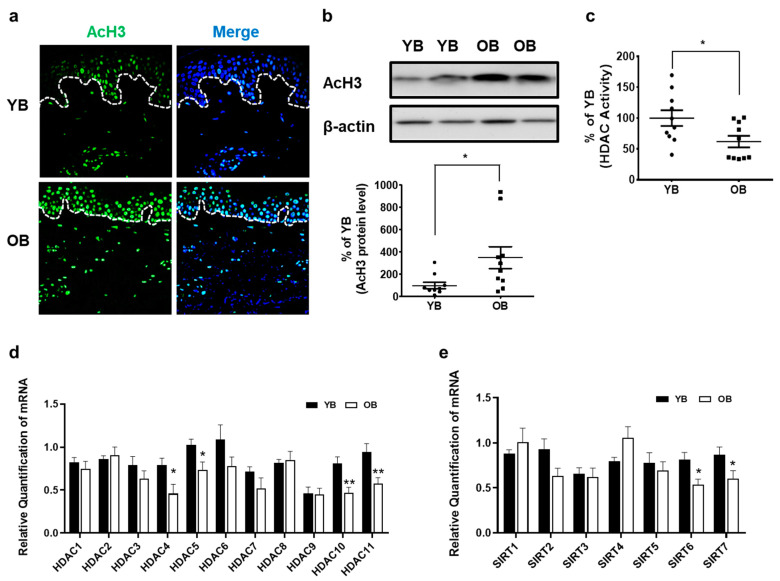
Acetylated histone H3 was increased, but HDAC activity and expression levels of HDAC4, HDAC5, HDAC10, HDAC11, SIRT6, and SIRT7 were decreased in intrinsically aged human skin in vivo. The buttock skin of young and elderly subjects was obtained by punch biopsy. Immunofluorescence analysis of AcH3 and DAPI staining of paraffin-embedded sections (*n* = 6). Original magnification: ×200 (**a**). Western blot analysis of AcH3 and β-actin. The bands are representative of the results obtained for 10 young and 10 elderly subjects (*n* = 10) (**b**). Total HDAC activity was measured using an HDAC assay kit (*n* = 10) (**c**). Expression levels of HDAC and SIRT mRNAs were compared in young (*n* = 10) and aged (*n* = 10) subjects by qPCR (**d**,**e**). Data were normalized to one of the young subjects. Data are presented as means ± SEM. Statistical significances were determined using a two-tailed Student’s *t*-test. * *p* < 0.05, ** *p* < 0.01 vs. YB. YB: young buttock skin; OB: old buttock skin.

To investigate the effects of intrinsic aging on AcH3 levels in vivo, the expression of AcH3 in sun-protected buttock skin was compared between young and elderly subjects. Immunofluorescence staining revealed that AcH3 expression was increased in both the epidermis and dermis of the elderly buttock skin, compared with that in the young buttock skin (Figure 2a). AcH3 levels, as observed by Western blot analysis, were significantly increased by 352.2% ± 86.4% in intrinsically aged buttock skin (Figure 2b). Global HDAC activity was significantly decreased by an average of 10.2% ± 3.7% in the skin from elderly subjects, compared with that in the skin from young subjects (Figure 2c). Therefore, our data indicate that the intrinsic aging process increases the expression of AcH3 and decreases HDAC activity in human skin in vivo.

We also investigated changes in the expression of all 11 HDACs and 7 SIRTs in the buttock skin of young and elderly subjects using qPCR. The expression of HDAC4, HDAC5, HDAC10, and HDAC 11 mRNAs was significantly decreased by an average of 42.8%, 28.4%, 42.2%, and 39%, respectively, in the buttock skin from elderly subjects, compared with that in the buttock skin from young subjects (Figure 2d). In addition, the expression of SIRT6 and SIRT7 mRNA was also significantly decreased by an average of 34.2% and 30.5%, respectively, in the buttock skin from elderly subjects, compared with that in the skin from the young subjects in vivo (Figure 2e).

### 2.3. Acetylation of Histone 3, HDAC Activity, and Expression of HDACs and SIRTs Do Not Differ between Photoaged Forearm Skin and Intrinsically Aged Buttock Skin

**Figure 3:**
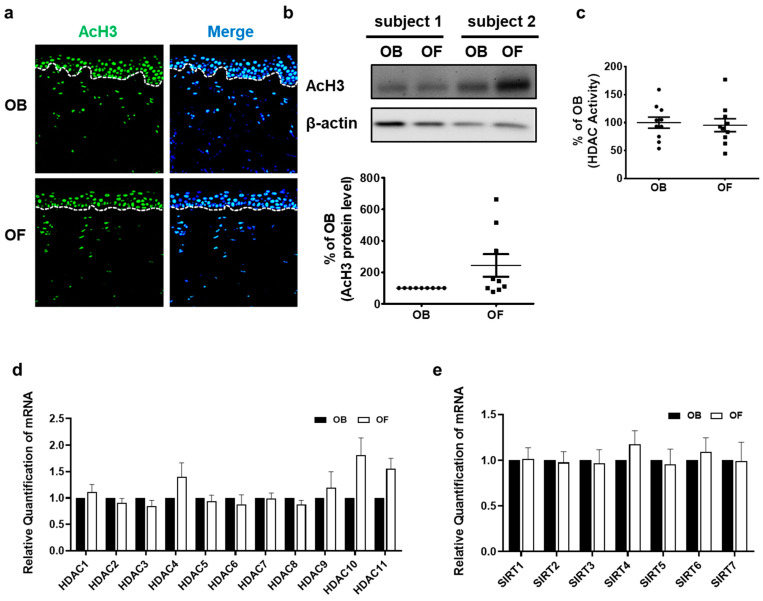
Acetylated histone H3, HDAC activity, and expression of HDACs and SIRTs did not show significant changes in photoaged human skin in vivo. Buttock and forearm skin of the same elderly subjects were obtained by punch biopsy. Immunofluorescence analysis of AcH3 and DAPI (*n* = 6). Original magnification: ×200 (**a**). Western blot analysis of AcH3 and β-actin protein (*n* = 9). The bands are representative of the results obtained in nine independent experiments (**b**). Total HDAC activity was measured using an HDAC assay kit in control or UV-irradiated human skin tissues (*n* = 10) (**c**). Expression levels of HDAC and SIRT mRNAs were compared in buttock and forearm skin of the same aged subjects by qPCR (*n* = 10) (**d**,**e**). Data were normalized to each individual’s buttock skin. The bar graph shows the means ± SEM. Statistical significances were determined using a two-tailed paired *t*-test. OB: Old buttock skin; OF: Old forearm skin.

To explore whether photoaging can further modulate histone acetylation and HDAC activity, AcH3 expression and HDAC activity were compared between sun-protected buttock skin and sun-exposed forearm skin of the same elderly individuals. No significant difference in AcH3 expression was observed between the sun-protected buttock and sun-exposed forearm skin by immunofluorescence staining (Figure 3a) and Western blot analysis (Figure 3b). Moreover, global HDAC activity in photoaged skin did not differ significantly from that in intrinsically aged skin (Figure 3c). Next, the expression of each HDAC and SIRT mRNA in the sun-protected buttock and sun-exposed forearm skin was examined. Consistent with similar global HDAC activity, there was no significant change in mRNA expression of any of the HDACs and SIRTs (Figure 3d,e) between sun-protected buttock and sun-exposed forearm skin. These findings suggest that photoaging does not additionally influence the expression of HDAC and the global histone acetylation status, compared with intrinsic aging.

## 3. Discussion

Epigenetic regulation is considered to play an important role in skin aging, but in vivo evidence for it is scarce, particularly in intrinsically aged and photoaged skin. Here, we investigated whether histone acetylation status, global HDAC enzyme activity, and HDACs and SIRTs expression characterize intrinsically aged, photoaged, and UV-irradiated skin in vivo.

In both acute UV-irradiated skin and intrinsically aged skin, the expression of AcH3 was increased, and global HDAC enzyme activity was decreased. This is in line with our previous finding that UV irradiation leads to increased histone acetylation and decreased total HDAC enzymatic activity in human skin fibroblasts [14]. In this study, we confirmed that UV irradiation is a stimulus that causes changes in histone modification in human skin in vivo.

We examined changes in the expression of all HDAC and SIRT enzymes in human skin in vivo. We found that HDAC4, HDAC11, and SIRT4 were significantly decreased in UV-irradiated skin, and HDAC4, HDAC5, HDAC10, HDAC11, SIRT6, and SIRT7 were significantly decreased in intrinsically aged skin. Notably, we found that the expressions of HDAC4 and HDAC11 were decreased both in acute UV-irradiated skin and intrinsically aged skin, suggesting their universal role in both processes. In a previous study wherein the mRNA expression of 11 HDACs and 7 SIRTs was examined by RNA-Seq analysis, an initial downregulation of HDAC members (HDAC4, HDAC7, HDAC9, and SIRT1) by UV was observed in human keratinocytes [21]. HDAC4 expression is dysregulated in several neurodegenerative diseases, such as Alzheimer’s and Parkinson’s diseases [22]. In addition, inhibition of HDAC4 led to senescence in human fetal lung fibroblasts [23]; silencing of HDAC4 expression impaired TGFβ1-induced α-SMA expression [24]; and inhibition of HDAC4 caused increased MMP-1 expression via the JNK pathway [25], suggesting that HDAC4 might be linked to the aging process. HDAC11 has functions in regulating pro- and anti-inflammatory responses in immune cells as well as in muscle differentiation [26,27,28], but little is known about the role of HDAC11 in skin aging. Further research is warranted to assess the role of HDAC4 and HDAC11 in skin aging.

We did not find a significant difference in the AcH3 level and HDAC enzyme activity in photoaged forearm skin and intrinsically aged buttock skin of the same elderly subject. In addition, the expression levels of HDACs and SIRTs did not show significant differences in photoaged skin. Our findings imply that the photoaging process does not cause further changes in histone H3 acetylation, global HDAC activity, and specific HDAC expression in intrinsically aged skin in vivo. In contrast to our findings, Ding et al. found that global histone H3 acetylation was increased in photoaged human epidermis and suggested that upregulated expression of the HAT gene, EP300, and downregulated expression of HDAC1 and SIRT1 might contribute to global histone H3 hyperacetylation in the sun-exposed epidermis [29]. The reasons for this discrepancy are not clear, but this might be attributable to the differences in the tissues used; Ding et al. only used the epidermis, whereas in the present study, we used the whole skin, including the dermis and epidermis. Besides, there has been a report suggesting a gender-dependent difference in methylation levels [30]. Further study is needed to identify whether histone acetylation in skin aging is also differentially regulated according to gender.

Anti-aging strategies target diverse mechanisms involved in skin aging, such as oxidative stress, mitochondrial dysfunction, cellular senescence, and epigenetic changes [1,31,32,33,34]. For example, melatonin and its metabolites, non-calcemic secosteroids, lumisterol derivatives, polyphenols, and vitamins represent promising anti-aging agents [1,35,36]. It has also been reported that natural products, such as epigallocatechin-3-gallate, genistein, curcumin, and resveratrol, can affect the activity of various epigenetic regulating enzymes, such as DNA methyltransferase, HATs, and HDACs [37,38]. Epigenetic modifications contribute to the aging process [1,31], and recent evidence suggests that epigenetic reprogramming may rejuvenate aged cells and tissues [39,40]. Anacardic acid or caffeic acid phenethyl ester may prevent skin aging as HAT inhibitors by downregulating UV-mediated acetylation of histone in human and mouse skin [41,42]. In contrast, our results suggest that an HDAC activator, particularly targeting HDAC4 and HDAC11, might be a potential anti-aging agent for skin.

In conclusion, we examined, for the first time, changes in the expression of all 11 HDAC and 7 SIRT genes in a set of skin exposed to acute UV irradiation, intrinsic aging, and photoaging conditions. Global HDAC activity and levels of specific HDAC and SIRT enzymes were significantly reduced in UV-irradiated human skin and intrinsically aged skin, which may account for increased histone acetylation. Based on our results, we suggest that epigenetic regulation that reduces histone acetylation by inducing the activity or expression of HDACs may improve UV-induced skin inflammatory responses and intrinsic skin aging.

## 4. Materials and Methods

### 4.1. Human Skin Samples

The buttock skin of 10 Korean volunteers without current or prior skin diseases (mean age, 43.8 y; range, 35–52 y) was irradiated with two minimal erythema doses (MEDs) of UV. MED is defined as the minimum amount of UV radiation required to produce minimal erythema. The irradiation intensity 20 cm from the light source was approximately 1.0 mW/cm^2^. A Waldmann UV-800 (Waldmann, Villingen-Schwenningenm, Germany) phototherapy device with a F75/85W/UV21 fluorescent lamp (emission range: 285–350 nm, peak at 310–315 nm) was used to irradiate sun-protected buttock skin. UV-C (<290 nm) was filtered by a Kodacel filter (TA401/407, Kodak, Rochester, NY, USA). In this study, UV irradiation included both UV-A and UV-B light. The MED on the buttock skin was determined 24 h after UV irradiation. The MED values ranged between 70 and 90 mJ/cm^2^ for the skin of Korean volunteers. At 24 h post-UV irradiation, non-irradiated and UV-irradiated skin samples were obtained. In addition, skin samples from sun-protected buttock skin and photodamaged forearm skin were obtained from elderly Korean volunteers (mean age 76.8 y; range 70–83 y) without current or prior skin diseases. This study was conducted according to the principles of the Declaration of Helsinki. All experimental procedures were approved by the Institutional Review Board at Seoul National University Hospital, and written informed consent was obtained from each subject (IRB No. 1410-133-621; 2014.12.08.).

### 4.2. Immunofluorescence Staining

Human skin samples were fixed in 10% formalin for 24 h. Thereafter, the samples were embedded in paraffin, and 4 μm sections were cut. The sections were then blocked with a blocking solution for 30 min at room temperature and incubated overnight with a primary rabbit polyclonal antibody against acetylated histone H3 (EMD Millipore, Billerica, MA, USA) in a humidified chamber at 4 °C. After washing in PBS, the sections were incubated with the secondary antibody, Alexa 488, at room temperature for 1 h. The sections were then washed with PBS and distilled water, and cell nuclei were counterstained with DAPI. The samples were mounted using Faramount Aqueous Mounting Medium (Dako, Carpinteria, CA, USA). The fluorescent images were acquired using a confocal microscope.

### 4.3. Histone Deacetylase Activity Assay

Global HDAC enzymatic activity was measured using an HDAC assay kit (Upstate Biotech, New York, NY, USA) according to the manufacturer’s protocol. Briefly, 30 µg of total protein extracted from human skin samples was incubated with the HDAC assay substrate for 60 min at 37 °C, which allowed deacetylation of the colorimetric substrate. The activator solution was then added to selectively release the colorimetric molecule from the deacetylated substrates and measured at 405 nm using a plate reader.

### 4.4. Western Blot Analysis

We obtained 4 mm punch biopsies of human skin. The tissues were physically disrupted and homogenized, and the protein lysates were isolated using RIPA buffer. The lysates were rotated at 4 °C for 15 min and centrifuged at 13,000 *g* for 15 min, and the supernatant was used for Western blot analysis. Western blotting was performed. Briefly, 50 μg of the protein samples was resolved on an 8–10% SDS-polyacrylamide gel and then transferred onto nitrocellulose membranes. An anti-acetylated histone H3 rabbit polyclonal antibody (Merck Millipore, Darmstadt, Germany) was used at a dilution of 1:1000 as the primary antibody. Immunoreactive proteins were visualized by enhanced chemiluminescence (Amersham, Buckinghamshire, UK). The band intensity was quantified using ImageJ software (National Institutes of Health, Bethesda, MD, USA), normalized to that of β-actin, and compared with that of the control group.

### 4.5. Quantitative Real-Time RT-PCR

Total RNA was isolated from human skin samples using RNAiso Plus (Takara Bio Inc., Shiga, Japan), and 2 μg of it was converted to cDNA using a First Strand cDNA Synthesis Kit (Thermo Fisher Scientific, Inc., Waltham, MA, USA) according to the manufacturer’s instructions. Quantitative PCR (qPCR) was performed using a 7500 Real-time PCR System (Applied Biosystems, Foster City, CA, USA) and SYBR Green PCR Master Mix (Takara Bio, Inc., Shiga, Japan). The primer sequences used are listed in Table 1. The PCR conditions were as follows: 50 °C for 2 min and 95 °C for 2 min, followed by 40 cycles at 95 °C for 15 s and 60 °C for 1 min. Data were calculated using the 2^−ΔΔCt^ method. Fold changes were calculated as the ratio of gene expression normalized to 36B4 in each group relative to that in the control group. These experiments were carried out in triplicate and were independently repeated at least three times.

### 4.6. Statistical Analysis

Data are presented as mean values ± standard error of the mean. When comparisons were made between two groups, statistical significances were determined using a two-tailed Student’s *t*-test for interindividual comparisons and two-tailed paired *t*-test for intraindividual comparisons. The statistical analysis was performed by Microsoft Excel 2016 (Microsoft Corporation, Redmond, WA, USA). *p*-values less than 0.05 were considered statistically significant.

## Figures and Tables

**Table 1:** Specific primers in used qPCR.

Gene	Primers (5′->3′)	
	Forward	Reverse
HDAC1 HDAC2 HDAC3 HDAC4 HDAC5 HDAC6 HDAC7 HDAC8 HDAC9 HDAC10 HDAC11 SIRT1 SIRT2 SIRT3 SIRT4 SIRT5 SIRT6 SIRT7 36B4	ATCTATCGCCCTCACAAAGC TGCTACTACTACGACGGTGA GAGAGTCAGCCCCACCAATA GAGAGACTCACCCTTCCCG ACAGCATGACCCCTGACAAGG ATCTGGCGGAGTGGAAGAA CTCACTGTCAGCCCCAGAG AAACGGGCCAGTATGGTG CAACAAAACCCTAGCAGCCT CACTAGCGAGGGCGTTTG GGATGCTACACACAACCCA GCGATTGGGTACCGAGATAA GACCCCTCTCACCCTCTG TCCCAGTTTCTTCTTTTCGAGTA GACAGGGTCCTGTGCTTG GGGGCCCAAGTAAATGGAAA TCCCGGAGATCTTCGACC TGTGGACACTGCTTCAGAAAGGGA TGGGCTCCAAGCAGATGC	AATCTCTGCATCTGCTTGCT AGTGGCTTTATGGGGCCTA GTTGTTCAGCTGGGTTGCTC CCGGTCTGCACCAACCAAG GCTCCTGCTGCCGCTTGG AAGTGACACTGGAGTCCTGA CTGGTGCTTCAGCATGACC CTGACCTTCTGGAGATGCTG GCCCACAGGAACTTCTGACT GGGTCGTCCCAGAGCA CCCATTTTCCGGCATCAAAG TTGCATGTGAGGCTCTATCC CAGGAAGTCCATGTCTGCTT GAAAGCTTCCCCTTGTCACT GCTCCTCTGAGAGAAAGACG TGAAGATGGTTGTCTCCACG CCAGAGGCAGTGCTGATG CACAGTTCTGAGACACCACATGCT GGCTTCGCTGGCTCCCAC

## Data Availability

The data that support the findings of this study are available from the corresponding author upon reasonable request.

## Abbreviations

AcH3	Acetylated histone H3
HDAC	Histone deacetylase
SIRT	Sirtuin
UV	Ultraviolet
